# Supplementary material for: Complexome profiling of the Chlamydomonas psb28 mutant reveals TEF5 as an early PSII assembly factor
Source: Plant Cell. 2025 Mar 17;37(6):koaf055. doi: 10.1093/plcell/koaf055 (PMC12164590; doi:10.1093/plcell/koaf055)
Supplement: koaf055_Supplementary_Data [file koaf055_supplementary_data.zip › psb28_interactive.zip!/plots/intensity/qualityPlots/peptides/SEC61A_Cre03.g171350.t1.2.html]

### Cre03.g171350.t1.2

Trivial name: SEC61A  
SeqCoverage [median]: 3.60  
Mass: 52.50 kDa  
  
 

| Sequence | MissedCleavage | Mass [Da] | wtIntensity | muIntensity | Score |
| --- | --- | --- | --- | --- | --- |
| DTTASLKK | 1 | 862.48 | 391.02 | 579.47 | 91.06 |
| GQQGNYPIK | 0 | 1003.51 | 295.56 | 635.83 | 114.60 |
|  |  |  | 686.6 | 1215.3 |  |
